# The Protective Role of Hyaluronic Acid in Cr(VI)-Induced Oxidative Damage in Corneal Epithelial Cells

**DOI:** 10.1155/2017/3678586

**Published:** 2017-03-27

**Authors:** Wei Wu, Hua Jiang, Xinnian Guo, Yu Wang, Shibo Ying, Lingfang Feng, Tao Li, Hailing Xia, Yixiao Zhang, Riping Chen, Tianhui Chen, Jianlin Lou

**Affiliations:** ^1^The Second Affiliated Hospital of Zhejiang University School of Medicine, Hangzhou, Zhejiang 310009, China; ^2^Institute of Occupational Disease, Zhejiang Academy of Medical Sciences, Hangzhou, Zhejiang 310013, China

## Abstract

Cr(VI) exposure could produce kinds of intermediates and reactive oxygen species, both of which were related to DNA damage. Hyaluronan (HA) has impressive biological functions and was reported to protect corneal epithelial cells against oxidative damage induced by ultraviolet B, benzalkonium chloride, and sodium lauryl sulfate. So the aim of our study was to investigate HA protection on human corneal epithelial (HCE) cells against Cr(VI)-induced toxic effects. The HCE cell lines were exposed to different concentrations of K_2_Cr_2_O_7_ (1.875, 3.75, 7.5, 15.0, and 30 *μ*M) or a combination of K_2_Cr_2_O_7_ and 0.2% HA and incubated with different times (15 min, 30 min, and 60 min). Our data showed that Cr(VI) exposure could cause decreased cell viability, increased DNA damage, and ROS generation to the HCE cell lines. But incubation of HA increased HCE cell survival rates and decreased DNA damage and ROS generation induced by Cr(VI) in a dose- and time-dependent manner. We report for the first time that HA can protect HCE cells against the toxicity of Cr(VI), indicating that it will be a promising therapeutic agent to corneal injuries caused by Cr(VI).

## 1. Introduction

Chromium (Cr) exists in many states, and the forms of hexavalent chromium [Cr(VI)] and trivalent chromium [Cr(III)] are found commonly in the environment. Cr(VI) is considered more toxic than Cr(III) as it can readily enter cellular membranes via nonspecific anion carriers. Cr(VI) is widely used in chemical industries such as electroplating, welding, dyes, paint pigments, leather tanning, and so forth. Many studies have reported that soluble hexavalent chromium is a powerful epithelial irritant with oxidizing and strong acid properties, leading to respiratory, dermatic, and ocular irritations, such as cough, dyspnea, sneezing, contact dermatitis, skin ulcers, eye redness, tearing, photophobia, and vision blurring [1–7].

It is widely accepted that cellular reduction of Cr(VI) is an activation process that generates variable intermediates [Cr(V) and Cr(IV)] and reactive oxygen species (ROS) [8–11]. The intermediates formed during cellular Cr(VI) reduction are able to cause hydroxyl radical generation that is responsible for DNA strand breaks [12]. Meanwhile, the comet assay has been found to be a very sensitive and reliable method for measuring DNA damage. ROS are generated as a by-product of normal mitochondrial activity and scavenged by an antioxidant system in aerobic cells. The balance between oxidants and antioxidants may be broken by overproduced ROS, which can cause oxidative stress such as lipid peroxidation (LPO), decreased cell viability, increased DNA damage, and apoptosis [13]. Oxidative stress can then play a major role in inducing genetic and epigenetic alterations to organisms and is associated with carcinogenesis [8, 9, 14–16]. Both in vivo and in vitro studies have reported that Cr(VI) exposure could induce oxidative stress, which is one of the mechanisms of toxicity of Cr(VI). Nevertheless, there are very limited data reporting the toxic effects and mechanisms of Cr(VI) on the eye.

The corneal epithelial cell is located in the outermost layer of the eyeball and is susceptible to oxidative damage. In the chemical manufacturing associated with chromium, occupational exposure increases the chance of damage on ocular surfaces such as the cornea. The cytotoxicity of ocular tissues induced by chromium was investigated rarely. Asmatullah and Shakoori studied the effect of hexavalent Cr on the development of chicken eye and found that in treated groups, the eye was defective with the undifferentiated cornea, lens, and retina [17]. Chromium picolinate could induce several hazards to the cornea and lens including a significant decrease in SOD, GSH, and Na^+^- and K^+^-ATPase levels; a significant increase in MDA level; and severe morphological and histological changes [13]. Apel et al. reported that cobalt-chromium toxicity could cause inner retinal dysfunction [18]. Hexavalent chromium-caused ocular trauma especially cornea injury has been reported in China [6, 7], while the toxicity and its mechanism are not very clear.

Hyaluronic acid (hyaluronan, HA) is a linear nonsulfated polysaccharide chain composed of *β*-1,4-glucuronic acid alternated with *β*-1,3-N-acetylglucosamine [19], which belongs to the glycosaminoglycan family. Hyaluronan, a ubiquitous extracellular matrix ingredient, exists in many parts of the human body such as the cornea, the vitreous body of the eye, joints, and skin. In clinical practice of ophthalmology, HA has impressive biological functions and is used in eye drops for dry eye syndrome to increase tear film stability and reduce subjective symptoms, such as ocular irritation and burning [20]. Hyaluronic acid (HA) is considered a remarkable antioxidant and ROS scavenger [21–23]. It was reported that high-molecular weight hyaluronan (HMW-HA, 1000 kDa) could protect corneal epithelial cells against oxidative damage induced by ultraviolet B [24, 25], benzalkonium chloride [22], sodium lauryl sulfate [26], and so on. Our study was performed to demonstrate whether HMW-HA is protective towards Cr(VI)-induced oxidative damage to corneal epithelial cells.

## 2. Materials and Methods

### 2.1. Cell Culture and Treatment

The human corneal epithelial cell line was provided by New York University. The cells were cultured in DMEM/F12 (Gibco, Grand Island, NY) supplemented with 10% fetal bovine serum (HyClone, USA), 5 *μ*g/ml insulin (Gibco), 0.1 *μ*g/ml cholera toxin, 5 ng/ml human epidermal growth factor (Gibco), and 40 *μ*g/ml gentamicin at 37°C in a fully humidified atmosphere with 5% CO_2_. The cells were subcultured every 2-3 days. Cells were exposed to potassium dichromate (K_2_Cr_2_O_7_, Sigma, USA) at concentrations of 1.875, 3.75, 7.5, 15.0, and 30 *μ*M or exposed to a combination of K_2_Cr_2_O_7_ and 0.2% HA (1000 kDa; Freda Biopharm Co. Ltd., Shandong, China) for 15 min, 30 min, and 60 min.

### 2.2. CCK-8 Assay

Cell viability was assessed by Cell Counting Kit-8 (CCK-8) (Dojindo Laboratories, Kumamoto, Japan), according to the description of our previous study [27]. Briefly, three wells were prepared for each sample in a 96-well plate. After treatment, 10 *μ*l of CCK-8 solution was added to each well and incubated at 37°C for 1 hour, and then, the optical density (OD) value for each well was measured at a wavelength of 450 nm on a microplate reader (Tecan Sunrise, Switzerland). The cell viability was calculated as follows:

(1) $$\begin{array}{c} Cell\;viability\left(\%\right)=\frac{OD\left(experiment\right)-OD\left(blank\right)}{OD\left(control\right)-OD\left(blank\right)}\times 100, \end{array}$$

where OD (experiment) is the absorbance of a well with treated cells and CCK-8, OD (blank) is the absorbance of a well with medium and CCK-8 but without cells, and OD (control) is the absorbance of a well with untreated cells and CCK-8.

### 2.3. Comet Assay

The alkaline comet assay was performed according to the description by Singh et al. [28]. The viability of cells was first assessed with the trypan blue dye exclusion test. Then, the cells were embedded in 0.65% low-melting point agarose at a final concentration of 10^4^ cells/ml; 75 *μ*l of this cellular suspension was then spread onto a frosted slide that had been previously covered with 100 *μ*l of 1% normal-melting point agarose (as the first layer). The slides were immersed in freshly prepared lysis solution (1% sodium sarcosinate, 2.5 M NaCl, 100 mM Na_2_EDTA, 10 mM Tris-HCl pH 10, 1% Triton X-100, and 10% DMSO) at 4°C for 1 h. Then, the slides were placed in a horizontal electrophoresis unit covered with fresh buffer (1 mM Na_2_EDTA, 300 mM NaOH pH 13) for 20 min. Electrophoresis was performed for 20 min at about 1.5 V/cm and 300 mA. Subsequently, the slides were washed gently 2 times in neutralization buffer (0.4 M Tris-HCl, pH 7.5). Each slide was stained with 40 *μ*l of ethidium bromide (20 *μ*g/ml). All the above steps were conducted under yellow light (580 nm) to avoid additional DNA damage.

Observations were made as previously described [27], using a fluorescence microscope (Olympus, BX51) equipped with a 530 nm excitation filter, a 590 nm emission filter, and a camera (Olympus, DP50). Fifty cells from each of the two replicate slides per sample were selected for data analysis, and the CASP software was used to analyze the comets and the percentage of DNA in the comet tail was calculated [29].

### 2.4. ROS Detection

The ROS generation in human B lymphoblastoid cells was measured with the 2′,7′-dichlorodihydrofluorescin diacetate (DCFH-DA) method [30] on the basis of the ROS-dependent oxidation of DCFH-DA to 2′,7′-dichlorofluorescin (DCF). After exposure to metal compounds, cells were washed twice and resuspended with PBS (1 × 10^6^ cells/ml). The suspended cells were incubated with DCFH-DA (5 *μ*M) at 37°C for 30 minutes. The fluorescence intensity of each sample was detected by a multimode plate reader at an excitation wavelength of 485 nm and an emission wavelength of 528 nm. Finally, the ratio between the fluorescence intensity of each treated sample over negative controls was calculated.

## 3. Results

### 3.1. Cell Survival

**Figure 1:**
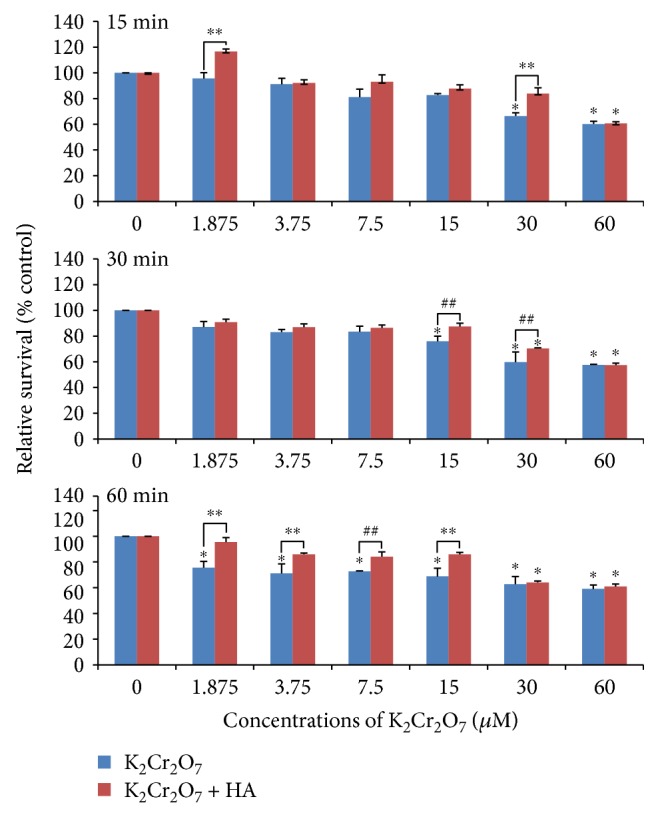
The effects of Cr(VI) or a combination of Cr(VI) and HA on the survival rates of HCE cells. ^∗^*p* < 0.01, compared with the control. ^∗∗^*p* < 0.01, ^##^*p* < 0.05, compared with the K_2_Cr_2_O_7_ group.

As shown in Figure 1, the cell survival rates of human corneal epithelial cells exposed to K_2_Cr_2_O_7_ alone decreased significantly (*p* < 0.01) at the concentrations of 30–60 *μ*M for the 15 min exposure group, 15–60 *μ*M for the 30 min exposure group, and 1.875–60 *μ*M for the 60 min exposure group, respectively. However, a significant (*p* < 0.01) decrease in relative cell survival rates of cells treated with a combination of K_2_Cr_2_O_7_ and 0.2% HA was only observed at the concentrations of 30–60 *μ*M, regardless of the exposure time. Moreover, 30 min preincubation of 0.2% HA could significantly (*p* < 0.01) enhance the relative cell survival rates of cells treated with K_2_Cr_2_O_7_ at the concentrations of 1.875 *μ*M or 30 *μ*M for the 15 min exposure group, 15–30 *μ*M for the 30 min exposure group, and 1.875–15 *μ*M for the 60 min exposure group, respectively.

### 3.2. DNA Damage

**Figure 2:**
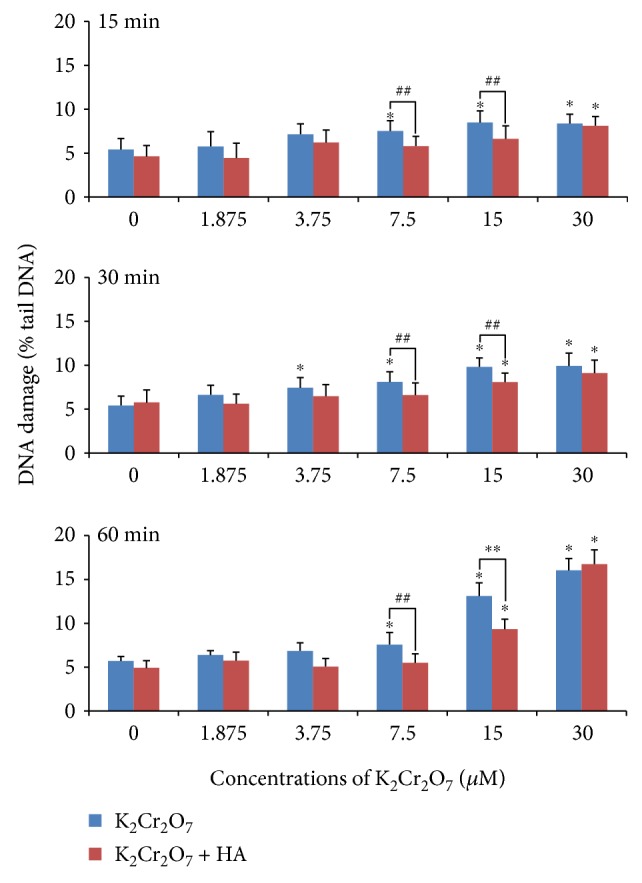
DNA damage induced by Cr(VI) or a combination of Cr(VI) and HA using the comet assay. ^∗^*p* < 0.01, compared with the control. ^∗∗^*p* < 0.01, ^##^*p* < 0.05, compared with the K_2_Cr_2_O_7_ group.

Figure 2 shows the results of DNA damage. A significant (*p* < 0.01) increase in DNA damage was induced by K_2_Cr_2_O_7_ at the concentrations of 7.5–30 *μ*M in all the three exposure groups (15 min, 30 min, and 60 min). But K_2_Cr_2_O_7_ combined with 0.2% HA could only cause DNA damage (*p* < 0.01 compared to the control group) at the concentration of 30 *μ*M in the 15 min exposure group and at the concentrations of 15–30 *μ*M in the 30 min and 60 min exposure groups. Furthermore, 30 min preincubation of 0.2% HA could significantly (*p* < 0.05 and *p* < 0.01) decrease DNA damage induced by K_2_Cr_2_O_7_ at the concentrations of 7.5 *μ*M and 15 *μ*M in all the three exposure groups (15 min, 30 min, and 60 min).

### 3.3. ROS Production

**Figure 3:**
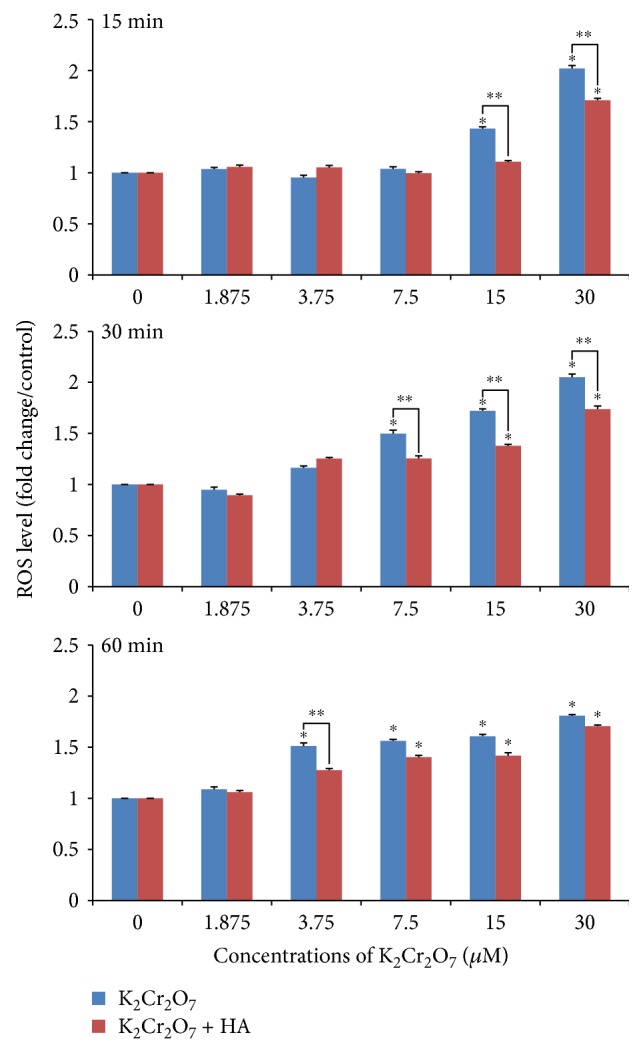
Cr(VI) or a combination of Cr(VI) and HA treatment induced ROS production in HCE cells. ^∗^*p* < 0.01, compared with the control. ^∗∗^*p* < 0.01, compared with the K_2_Cr_2_O_7_ group.

The results of ROS generation are shown in Figure 3. A dose- and time-dependent increase of ROS levels was observed in cells treated with K_2_Cr_2_O_7_ alone, especially at the concentrations of 15–30 *μ*M, 7.5–30 *μ*M, and 3.75–30 *μ*M in the 15 min, 30 min, and 60 min exposure groups (*p* < 0.01), respectively. Preincubation of 0.2% HA could significantly weaken the effects of K_2_Cr_2_O_7_ on ROS generation, and statistically significant (*p* < 0.01) difference of ROS production was observed at the concentrations of 15–30 *μ*M, 7.5–30 *μ*M, and 3.75 *μ*M in the 15 min, 30 min, and 60 min exposure groups, respectively.

## 4. Discussion

Most of the previous studies on hexavalent chromium focused on its carcinogenic effects, and various kinds of cell lines related to the respiratory system were used in in vitro studies. However, the toxic effects of hexavalent chromium on ocular surface cells were rarely investigated. Actually, workers occupationally exposed to hexavalent chromium also have many chances of exposing their ocular surface to this kind of chemicals and thus suffer damage to their ocular surface cells. Such cases have been reported in Chinese literatures. What is more, the epithelial corneal cells located on the most external cellular layers of the ocular surface are the first line to encounter environmental insults and play an important role in protecting the inner ocular tissues. Therefore, the present study investigated the cytotoxic effects, DNA damage, and ROS generation induced by hexavalent chromium in a human corneal epithelial cell line. Furthermore, the protective role of hyaluronic acid against toxic effects induced by hexavalent chromium was also studied.

The alkaline comet assay is a sensitive method for direct visualization of DNA strand breakage at the level of single cells. DNA damages have been reported in many Cr(VI) exposure studies [31]. DNA strand breaks, which belong to one of these DNA damages, have been reported in various kinds of cells using the comet assay [32–36]. And it is recognized that DNA strand break is caused by the reduction of Cr(VI) to lower oxidation states instead of Cr(VI) [12]. Our results show a clear concentration- and time-related increase in DNA damage by Cr(VI), which were consistent with the previous study conducted in human B lymphocytoid cells by Lou et al. [36]. The mechanism of DNA damage induced by Cr(VI) may be partially attributed to the production of ROS.

Our results indicated that K_2_Cr_2_O_7_ could induce ROS in human corneal epithelial cells even after 15 min exposure and the ROS levels increased after K_2_Cr_2_O_7_ treatment in a time- and dose-dependent manner. Similar results were reported in previous studies conducted in human liver carcinoma (HepG2) cells [35] or in human B lymphoblastoid cells [36], but the exposure time of Cr(VI) in these studies was usually longer than 1 h. More recently, Lee et al. [37] demonstrated that a significant increase in ROS level was observed in HaCaT cells exposed to 15 *μ*M of Cr(VI) for less than 1 h, and it was in accordance with our results. Overproduced ROS, which include superoxide, hydrogen peroxide, hydroxyl radicals, and so on, can attack important biomolecules such as DNA, proteins, and lipids [38]. For example, Atilano et al. found that hydrogen peroxide could cause mitochondrial DNA damage in corneal epithelial cells [39]. Therefore, we speculated that the balance between an antioxidant system and ROS generation was destroyed by the surplus ROS produced by Cr(VI), then leading to DNA damage.

Hyaluronic acid, because of its unique molecular structure and physicochemical properties, plays important physiological functions in the body, such as lubricating joints, regulating vascular wall permeability, regulating the diffusion and transport of proteins and electrolytes, and promoting wound healing. There have been literatures reported that a preincubation of HMW-HA could protect corneal epithelial cells against oxidative damage induced by ultraviolet B [24, 25], benzalkonium chloride [22, 40], EDTA [41], sodium lauryl sulfate [26], and so on. In the present study, we confirmed the protective role of 0.2% 1000 kDa HA against genotoxicity of Cr(VI) on HCE cells. HA preincubation effectively enhanced the relative cell survival rates and reduced the DNA damage and ROS generation induced by Cr(VI) in HCE cells. It could be explained that HA may serve as a scavenger of free radicals and as an antioxidant, which can potentially absorb ROS [22]. Moreover, it was considered that HA could specifically bind to some cell surface receptors, such as CD44 receptor, which has been proved to be expressed on human epithelial cells [22]. Their study suggested that HA could protect cell membranes by interacting with the CD44 receptor. We thought that the CD44 receptor could be a key to understanding how HMW-HA protects HCE cells from genotoxicity of Cr(VI), and we would like to consider such research.

In conclusion, our present study showed that Cr(VI) could increase ROS formation and cause DNA strand breaks in HCE cells. In addition, 1000 kDa HA significantly reduces all the Cr(VI)-induced cytotoxic effects we observed. A study [22] suggested that 1000 kDa HA could form a cytoprotective coat on the cell membrane and thus prevents BAK cytotoxicity. HA (1000 kDa) may be a potential therapeutic agent to corneal injuries caused by Cr(VI) and other toxic agents. However, in vitro studies may not reflect the real situations in vivo, so further in vivo studies are needed to extrapolate these in vitro findings to clinical applications.
